# Oxidative stress levels and dynamic changes in mitochondrial gene expression in a radiation-induced lung injury model

**DOI:** 10.1093/jrr/rry105

**Published:** 2018-12-26

**Authors:** Zhongyuan Yin, Guanghai Yang, Sisi Deng, Qiong Wang

**Affiliations:** 1 Cancer Center, Union Hospital, Tongji Medical College, Huazhong University of Science and Technology, Wuhan, China; 2 Department of Thoracic Surgery, Union Hospital, Tongji Medical College, Huazhong University of Science and Technology, Wuhan, China

**Keywords:** radiation-induced lung injury, biopsy, ROS, mtDNA, mitochondrial respiratory complex

## Abstract

The purpose of this study was to set up a beagle dog model, for radiation-induced lung injury, that would be able to supply fresh lung tissues in the different injury phases for research into oxidative stress levels and mitochondrial gene expression. Blood serum and tissues were collected via CT-guided core needle biopsies from dogs in the various phases of the radiation response over a 40-week period. Levels of reactive oxygen species (ROS) and manganese superoxide dismutase 2 (MnSOD) protein expression in radiation-induced lung injury were determined by *in situ* immunocytochemistry; malondialdehyde (MDA) content and reductase activity in the peripheral blood were also tested; in addition, the copy number of the mitochondrial DNA and the level of function of the respiratory chain in the lung tissues were assessed. ROS showed dynamic changes and peaked at 4 weeks; MnSOD was mainly expressed in the Type II alveolar epithelium at 8 weeks; the MDA content and reductase activity in the peripheral blood presented no changes; the copy numbers of most mitochondrial genes peaked at 8 weeks, similarly to the level of function of the corresponding respiratory chain complexes; the level of function of the respiratory chain complex III did not peak until 24 weeks, similarly to the level of function of the corresponding gene *Cytb*. Radiation-induced lung injury was found to be a dynamically changing process, mainly related to interactions between local ROS, and it was not associated with the levels of oxidative stress in the peripheral blood. Mitochondrial genes and their corresponding respiratory chain complexes were found to be involved in the overall process.

## INTRODUCTION

Approximately 60% of all cancer patients receive radiotherapy at some point during the course of their disease, and late toxicity to normal tissues limits the radiation dose that can be applied to the tumor. Moreover, tolerable doses are often linked to suboptimal tumor control, in addition to side effects that lead to decreased quality of life; for example, radiation-induced pneumonitis and fibrosis are dose-limiting side effects of thoracic irradiation [1–4]. Although considerable progress has been made during the last decade with respect to the discovery of the involved effector cells and soluble mediators, the determination of the network of pathophysiological events and the cellular cross-talk that links acute tissue damage to chronic inflammation and fibrosis still requires further investigation.

In recent years, it has been shown that cellular oxidative stress plays an important role in radiation-induced lung injury (RILI), and thus, oxidative stress can be used as a monitoring index for the development, progression and prognosis of RILI [5]. A key factor in determining the state of oxidative stress is the presence of reactive oxygen species (ROS), which can interact with bases, ribose and phosphodiester linkages and can cause deoxyribonucleic acid (DNA) damage and subsequent apoptosis [6]. A growing number of studies have demonstrated that mitochondria are important sites for ROS production in cells, and that the exact location of the production is the substrate end of the respiratory chain. Under normal physiological conditions, only a small amount of ROS is produced, but when the mitochondrial respiratory chain is disturbed, many types of respiratory chain enzyme complexes will oxidize to produce large amounts of ROS [7]. The mitochondria are extremely susceptible to external factors, and radiation is the most common cause of ROS induced by damage to the mitochondrial respiratory chain [8].

Manganese superoxide dismutase 2 (MnSOD) is a superoxide dismutase that protects lung tissue and reduces RILI. Mitochondria are the main functional target of MnSOD. MnSOD blocks cell damage by maintaining mitochondrial DNA (mtDNA) stability [9]. In mouse models of lung injury, it has been confirmed that the levels of selective expression of mitochondrial MnSOD in alveolar Type II epithelial cells and vascular endothelial cells increase simultaneously [10]. Therefore, MnSOD is also an important factor in studies of RILI caused by mtDNA mutations.

Unlike nuclear DNA mutations, mtDNA mutations are heterogeneous. Because each cell contains hundreds or thousands of copies of mtDNA, each time a mutation occurs, wild-type and mutant mtDNA co-exist in cells, which results in so-called mtDNA heterogeneity [11].This heterogeneity also exists in normal individuals and is very predominant [12]. Given that the initial states of mitochondria differ among individuals, each person may exhibit different responses to radiation. [13]. Acute pneumonitis was observed the first 3 months after radiation, and chronic lung fibrosis was found during the next 4–12 months in all the dogs exposed to radiation. Computed tomography (CT)-guided core needle lung lesion biopsies were extracted from each dog four times over the course of one year. Here, we aim to explore the possible mechanisms of RILI by observing the changing dynamics in the mtDNA copy numbers and in the function of the corresponding respiratory chain complexes, as well as the levels of local and systemic oxidative stress markers at different stages of RILI.

## MATERIALS AND METHODS

### Animals

We purchased and bred beagle dogs from the Department of Veterinary Clinical Sciences of Tongji Medical College, Huazhong University of Science and Technology. At the time of purchase, they were 19–23 months old and weighed approximately 15 kg. All of the dogs underwent a physical examination. All studies were performed according to a protocol approved by the Huazhong University of Science and Technology Institutional Animal Care and Use Committee.

### Radiation

The dogs were randomly assigned into two groups. Three dogs received 18 Gy X-ray (Priums-k, SIEMENS) radiation in one fraction. One dog was never irradiated and was used as a control (Table 1). Approximately two-thirds of the right lung of each dog was exposed to radiation fields ranging from 7 × 14 to 8 × 17 cm. To protect the heart and spinal cord, we restricted 65–70% of the right lung volume, which was covered by the 95% prescription isodose line (Table 1) [13].

**Table 1.** Actual and fractional volumes of right lung and heart irradiation

		Right lung volume (cm^3^)	Fractional volume of right lung covered by 95% prescription isodose line (cm^3^)/(%)	Fractional volume of right lung covered by 50% prescription isodose line (cm^3^)/(%)	Heart volume (cm^3^)	Fractional volume of heart covered by 95% prescription isodose line (cm^3^)/(%)	Fractional volume of heart covered by 50% prescription isodose line (cm^3^)/(%)
**Control**	Dog1	288			178		
**18 Gy in 1 fraction**	Dog2	275	179/65	254/92	178	5.34/3	30.3/17
	Dog3	280	196/70	260/93	173	3.46/2	34.6/20
	Dog4	290	194/67	267/92	175	5.25/3	28.0/16

### CT scan

Weekly low-dose CT scans of the dogs were performed until 4 weeks after the first exposure to the radiation. The interval increased to 2 weeks from 4 weeks to 8 weeks, to 4 weeks from 8 weeks to 24 weeks, and to 8 weeks from 24 weeks to 40 weeks (Table 2). CT scans were performed using a third-generation scanner (Siemens Balance) set at 0.5-cm slice thickness, 130 kVp, 90 mAs and a high-resolution algorithm. The dogs were scanned in sternal recumbency 10 min after administration of pentobarbital sodium (Amersco, USA).

**Table 2.** Schedule of CT scan surveillance, radiation and CT-guided needle biopsy for the 4 dogs

		1 w-Pre	0 w	1 w	2 w	3 w	4 w	6 w	8 w	12w	16w	20w	24w	32w	40w
**Control**	Dog1	CTS, Biop	CTS,	CTS,	CTS,	CTS,	CTS, Biop	CTS,	CTS, Biop	CTS,	CTS,	CTS,	CTS, Biop	CTS,	CTS,
**18 Gy in 1 fraction**	Dog2	CTS, Biop	CTS, Rad	CTS,	CTS,	CTS,	CTS, Biop	CTS,	CTS, Biop	CTS,	CTS,	CTS,	CTS, Biop	CTS,	CTS,
	Dog3	CTS, Biop	CTS, Rad	CTS,	CTS,	CTS,	CTS, Biop	CTS,	CTS, Biop	CTS,	CTS,	CTS,	CTS, Biop	CTS,	CTS,
	Dog4	CTS, Biop	CTS, Rad	CTS,	CTS,	CTS,	CTS, Biop	CTS,	CTS, Biop	CTS,	CTS,	CTS,	CTS, Biop	CTS,	CTS,

w = week, CTS = CT Scan, Rad = radiation, Biop = biopsy.

### Sample collection and biopsy

All of the animals were clinically monitored 24 h after each biopsy, depending on the clinical signs and severity of any complication. Blood serum was collected from the dogs in various phases of the radiation response. Biopsy was performed once a week prior to the first exposure of radiation, and 4, 8 and 24 weeks after the radiation exposure over the course of one year (Table 2) [13].

### *In situ* determination of ROS indicators, including superoxide anion, hydrogen peroxide and hydroxyl free radicals

The expression of superoxide anion in lung tissues was detected by fluorescence probe dihydroethidium bromide (DHE) *in situ* on frozen sections of lung tissues. The expression of hydrogen peroxide and hydroxyl free radicals in lung tissues was detected by fluorescence probe 6-chloromethyl-2′,7′-dichlorodihydrofluorescein diacetate, acetyl ester (CM-H2DCFDA) *in situ* on frozen sections of lung tissues. DHE and CM-H2DCFDA can pass through the cells membrane freely, and they would have stayed in the cells for a long time. Once they are oxidized, they can emit fluorescence [14].

Frozen sections of fresh lung tissues were prepared at a 10 μm thickness. Under room temperature conditions, 100 μl of pre-cooled cleansing solution was added to slices so to cover the entire tissue surface. Then, the cleansing liquid was removed and replaced with 50 μl of working solution (stain) pre-warmed at room temperature. The sections were kept in an incubator at 37°C for 20 min, and then the working solution was removed, followed by the addition of 100 μl of cleansing solution. After removal of the cleansing solution, the sections were then sealed onto the slides by the addition of 10 μl of anti-fluorescence quenching solution. The tissues were observed immediately under a confocal laser microscope with an excitation wavelength (Lasers) of 543 nm, 30.0% maximum output power and emission wavelengths (Filters) of 561–681 nm. Sections were examined at a magnification of ×200, and images were acquired by ZEN 2009 software.

### Immunohistochemical detection of MnSOD expression in lung tissues of beagles

Fresh lung tissue specimens were cut into 4 μm-thick sections, following tissue fixation in 4% paraformaldehyde and subsequent paraffin embedding. A rabbit polyclonal anti-MnSOD antibody (1:400) and a horseradish peroxidase (HRP)-conjugated goat anti-rabbit secondary antibody were used to stain the tissues prior to microscopic examination. With respect to the controls, both positive and negative controls were established for each experiment. For positive controls, known MnSOD-positive sections from beagles were used; for the negative controls, we used phosphate-buffered saline (PBS) solution instead of the primary antibody. The immunohistochemical evaluation of MnSOD was performed by assigning scores for staining intensity, as follows: colorless was scored as 0, light yellow as 1, brownish yellow as 2 and brown as 3 [15].

### Detection of malondialdehyde content in the plasma by the thiobarbituric acid method

ROS can attack polyunsaturated fatty acids on cell membranes, and then cause lipid peroxidation, forming lipid peroxides such as malondialdehyde (MDA). Therefore, the content of MDA can reflect the level of lipid peroxidation in the body, and thus indirectly reflect the degree of cell damage. The condensation reaction of MDA with thiobarbituric acid (TBA) results in the formation of red products with a maximum absorption peak at 532 nm.

Undiluted plasma was obtained from each group of beagles and was fully mixed in selected centrifuge tubes with screw-on caps. The samples were incubated in a water bath at 95°C for 80 min. The tubes were heated carefully to avoid the splashing of liquid. After the tubes were removed from the incubator, they were immediately cooled in cold water before they were centrifuged at 4000 rpm for 20 min. The samples were then transferred from each test tube to 3 wells of a 96-well plate, with 300 μl in each well. The multifunctional microplate reader was calibrated at 532 nm, and the absorbance (OD) of each well was measured. The MDA content in the plasma was calculated from the OD values using the formula below.

$$\begin{array}{c} \mathrm{Plasma MDA content}\left(\mathrm{nmol}/\mathrm{ml}\right)\\ =\frac{\left({\mathrm{OD}}_{\mathrm{value of the tube containing the test sample}}{-\mathrm{OD}}_{\mathrm{value of the blank tube}}\right)}{\left({\mathrm{OD}}_{\mathrm{value of the tube containing the standard solution}}{-\mathrm{OD}}_{\mathrm{value of the blank tube}}\right)}\\ \times \mathrm{Concentration of standard solution}\left(\mathrm{10 nmol}/\mathrm{ml}\right) \end{array}$$

### Detection of the activities of plasma total SOD, CuZnSOD and MnSOD using a xanthine oxidase assay, and the detection of plasma GSHPX activity using an enzymatic biochemical method

Superoxide dismutase (SOD) can scavenge superoxide anion (O_2_^–•^) and protect cells from damage. The reaction system of xanthine oxidase produces O_2_^–•^, which can oxidize hydroxylamine to form nitrite, and then react with a chromogenic agent to form a purple-red product. If SOD is detected in the samples, it specifically inhibits the production of O_2_^–•^. The OD value of the measuring tube is lower than the OD value of the control tube, and the activity of SOD in the tested sample can be calculated by the formula. There are only two kinds of SOD in beagle dog cells, so the activity of MnSOD added to that of CuZnSOD, is the activity of the total SOD (T-SOD) [16].

T-SOD and CuZuSOD are both detected using the same xanthine oxidase method. Undiluted plasma was obtained from beagles and was pre-assessed by diluting it in a 0.9% saline solution at a ratio of 1:1 or 1:4. The inhibition rates of T-SOD of undiluted, 1:1 diluted and 1:4 diluted plasma samples were calculated to be 48.1%, 28.3% and 13.5%, respectively. When the inhibition rate is between 45% and 50%, the corresponding plasma concentration is best for detection, i.e., undiluted plasma is the best for detection. Therefore, 50 μl of an undiluted plasma sample was thoroughly mixed with the appropriate reagents provided with the detection kit (Nanjing Jiancheng Bioengineering Institute, China) before the sample was centrifuged at 4000 rpm for 15 min. The supernatant was collected to examine the CuZuSOD activity. Three 300-μl aliquots were taken from each test tube and were added to a 96-well plate. The OD value was determined at a wavelength of 550 nm by a multifunctional microplate reader. The method used to detect glutathione peroxidase (GSHPx) activity was the same as that mentioned above, but the OD value was read at 412 nm.

The following formula was used to calculate the inhibition rate:

$$\begin{array}{c} \mathrm{Inhibition rate}\\ =\frac{\left(\mathrm{OD value of T}\text{-}\mathrm{SOD control}\text{-}\mathrm{OD value of T}\text{-}\mathrm{SOD test sample}\right)}{\left(\mathrm{OD value of T}\text{-}\mathrm{SOD control}\right)}\\ \times \mathrm{100}\% \end{array}$$

$$\begin{array}{c} \mathrm{Plasma T}-\mathrm{SOD activity}\left(U/\mathrm{ml}\right)\\ =\frac{\left(\mathrm{OD value of T}\text{-}\mathrm{SOD control}\text{-}\mathrm{OD value of T}\text{-}\mathrm{SOD test sample}\right)}{\left(\mathrm{OD value of T}\text{-}\mathrm{SOD control}\right)}\\ ÷\mathrm{50}\%\times \text{dilution factor of the reaction system} \end{array}$$

The method used to determine the CuZnSOD activity in the plasma was the same as that used for T-SOD, as described above.

The plasma MnSOD activity (U/ml) = Plasma T-SOD Activity (U/ml)−Plasma CuZnSOD Activity (U/ml)

## Detection of the changes in mitochondrial gene copy numbers in lung tissues by quantitative real-time polymerase chain reaction

The DNA in treated cells and tissues was extracted, and the copy numbers of each gene in the mtDNA were examined by quantitative real-time polymerase chain reaction (qPCR). Using the coding sequences (CDSs) published in the GenBank database as a reference, we designed primers using Beacon Designer 7 primer design software to produce 80–150 bp of DNA amplification products (Table 3). The specificity of the primer sequences was confirmed using BLAST. The primers were synthesized and purified using DSL from Invitrogen (Shanghai, China). The copy numbers of each mitochondrial gene were calculated according to the method described below. The mitochondrial genes *12S rRNA*, *ND4*, cytochrome b (*Cytb*), cyclooxygenase 2 (*Cox2*) and adenosine triphosphate (ATP) 6 were the target genes, the expressions of which were compared against that of the base reference gene *GAPDH* [17]. The tissues and cells were irradiated at 18 Gy, and DNA samples were obtained at 0 h, 4 weeks, 8 weeks and 12 weeks. The 0 h group served as the control, while samples from the other time points served as the test groups. The changes in the mitochondrial gene copy numbers were determined using the comparative CT method (ΔΔCt). The amplification curve and the product melting curve were further analyzed after the qPCR reaction.

$$\begin{array}{c} \Delta \Delta \mathrm{Ct}={\left({\mathrm{Ct}}_{\mathrm{target genes}}{-\mathrm{Ct}}_{\mathrm{reference gene}}\right)}_{\mathrm{test group}}\\ \text{−}{\left({\mathrm{Ct}}_{\mathrm{target gene}}{-\mathrm{Ct}}_{\mathrm{reference gene}}\right)}_{\mathrm{control group}} \end{array}$$

**Table 3.** Primers of lung tissue mitochondrial genes

Gene name	Sequence (5′–3′)
dog*GAPDH-F*	TCTCTGCTCCTTCTGCTGATG
dog*GAPDH-R*	CCTGCCATACCTACCTGACAAT
dog*12srRNA-F*	GCCAGAGGACTACTAGCAATAGC
dog*12srRNA-R*	TTAGCGAAAGGTGGTGAGGTTT
dog*ND4-F*	CTGGTTATCGTAGCGGTTCTT
dog*ND4-R*	GGATTCGTTCGTAATTGGAGTTG
dog*Cytb-F*	AACCATAGCCACAGCATTCAT
dog*Cytb-R*	CGCCTCAGATCCATTCTACTAAG
dog*Cox2-F*	GCCGTTCCATCACTAGGTCTA
dog*Cox2-R*	AGTCCTGGTCGTATGGCTATAAG
dog*ATP6-F*	TGGCTCAACTAATCTACTTGGA
dog*ATP6-R*	GTGCTAAGGATGCTTTGGTTT

### Isolation of mitochondria

Lung mitochondria were isolated according to standard procedures with minor modifications. Briefly, lung homogenates were incubated in Sodium Chloride-Tris-EDTA (STE) buffer containing sucrose (250 mM), Tris pH 7.4 (10 mM), and ethylene glycol-bis-(b-aminoethyl ether)-N, N9-tetraacetic acid (EGTA) (2 mM), followed by centrifugation (1000 × 3*g*) for 3 min at 48°C. Supernatants were then collected and centrifuged (10 000 × 3*g*) for 10 min at 48°C. The resultant pellets were suspended in STE buffer and centrifuged (10 000 × 3*g*) for 10 min at 48°C. The pink and light brown pellets surrounding the dark brown center were aspirated, and the remaining pellet was suspended in STE buffer (1 ml) and centrifuged (10 000 × 3*g*) for 15 min at 48°C. Finally, the cell pellet was suspended in potassium phosphate buffer and stored at –80°C [18].

### Assays to evaluate respiratory complexes

We operated the assays under the protocols from the reagent kits (Wuhan, China, Bosk). To prepare for the measurement of respiratory chain complex enzymes, the mitochondrial suspension was diluted to 2 mg/ml and stored as aliquots at −70°C. Before measurements were obtained, the samples were freeze-thawed three times to fragment the mitochondrial membrane. Then, the expression levels of respiratory chain complexes I, II, III and IV and ATP synthase were examined by spectrophotometry according to the manufacturer’s instructions. The activity unit of the respiratory chain complexes is nmol/(min·mg) protein, while the activity unit of ATP synthase is μmol Pi/(min·mg).

### Statistical analyses

For each experiment, samples were isolated and pooled from groups of beagle dogs (*n* = 3), and all conditions were studied at the same time. One-way ANOVA, the Tukey–Kramer Multiple Comparisons test (for multiple groups), or Student’s *t*-test (for comparisons between two groups) were used. *P* < 0.05 was considered to be statistically significant.

## RESULTS

### Dynamic changes in CT scanning, pathological transformation and tissue acquisition of the lungs of beagles

We observed no significant changes in the body temperature, body weight, mental status, intake of food and water or in urination and defecation between the irradiated groups and the non-irradiated groups of dogs during the 40-week period. In the lungs of beagles, the changes in the CT images after exposure to radiation occurred only in the irradiated area, and no lesions were observed in the contralateral areas. Four weeks after exposure to 18 Gy of radiation, the appearance of the lung tissues changed to a ‘frosty-glass’ appearance, with increased patchy density and imaging shadows. Through hematoxylin–eosin (HE) staining, it was shown that the alveolar space became smaller, the alveolar wall was thickened, the numbers of fibroblasts and capillaries in the wall were increased, and a large number of inflammatory cells could be seen by light microscopy. Eight weeks after irradiation, large areas within the lung tissues became consolidated, normal air-containing lung tissues were mostly lost, and visible signs of bronchitis were observed. Alveolar walls were thickened, whereas the alveoli shrank significantly. Moreover, the amount of fiber and the number of fibroblasts increased, and both the numbers and sizes of Type II alveolar epithelial cells increased, along with the appearance of fibrin-like substances. Twenty-four weeks after irradiation, the local alveolar walls were completely replaced with collagen, most of the alveoli had collapsed or had even disappeared, and the number of cells was diminished [13]. Each time we were able to obtain 25–30 μg of lung-tissue specimens from the site of exposure using CT-guided techniques for the lung biopsy.

### Dynamic changes in reactive oxygen species and MnSOD protein expression in radiation-induced lung injury in beagles by *in situ* immunocytochemistry

#### Dynamic changes in the amount of superoxide anion, hydrogen peroxide and hydroxyl free radicals

**Fig. 1.**
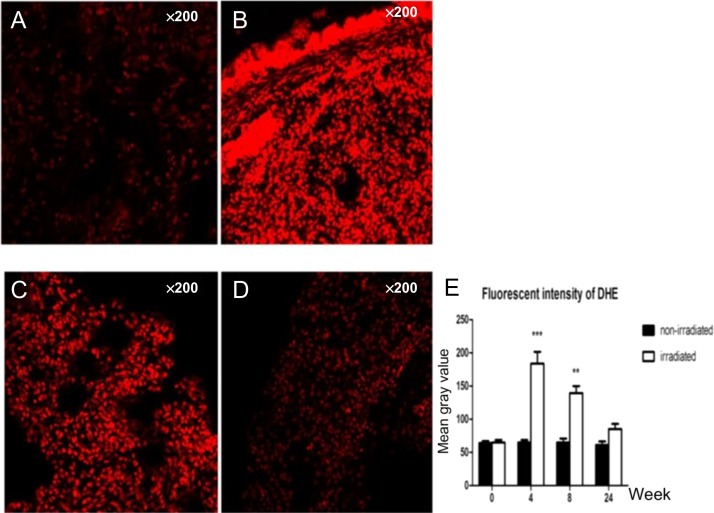
Dynamic changes in the amount of superoxide anion: (A) only very small amounts of superoxide anion showed in the control group; (B) 4 weeks after radiation; (C) 8 weeks after radiation; and (D) 24 weeks after radiation. (E) Compared with the control group, the irradiated samples showed dynamic changes, peaking at 4 weeks, then gradually decreasing over time. At 4 weeks and 8 weeks, the *P* values were both <0.05.

**Fig. 2.**
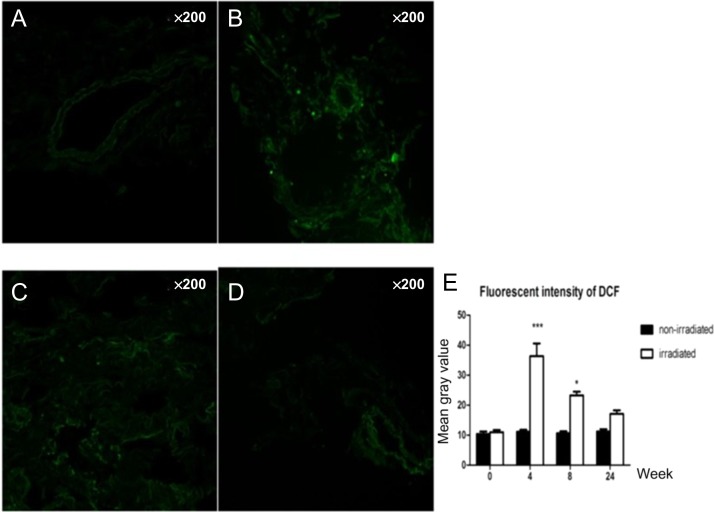
Dynamic changes in the amount of hydrogen peroxide and hydroxyl free radical: (A) only very small amounts of hydrogen peroxide and hydroxyl free radical showed in the control group; (B) 4 weeks after radiation; (C) 8 weeks after radiation; and (D) 24 weeks after radiation. (E) Compared with the control group, the irradiated samples showed dynamic changes, peaking at 4 weeks, then gradually decreasing over time. At 4 weeks and 8 weeks, the *P* values were both <0.05.

The amounts of superoxide anion, hydrogen peroxide and hydroxyl free radicals in the irradiated groups were higher than those in the non-irradiated groups. Compared with the control groups, the irradiated samples showed dynamic changes and peaked at 4 weeks, which was followed by a gradual decrease over time. At 4 weeks and 8 weeks, *P* values were both <0.05 (Figs 1 and 2).

#### Dynamic changes in MnSOD protein expression in lung tissues of beagles with radiation-induced lung injury

**Fig. 3.**
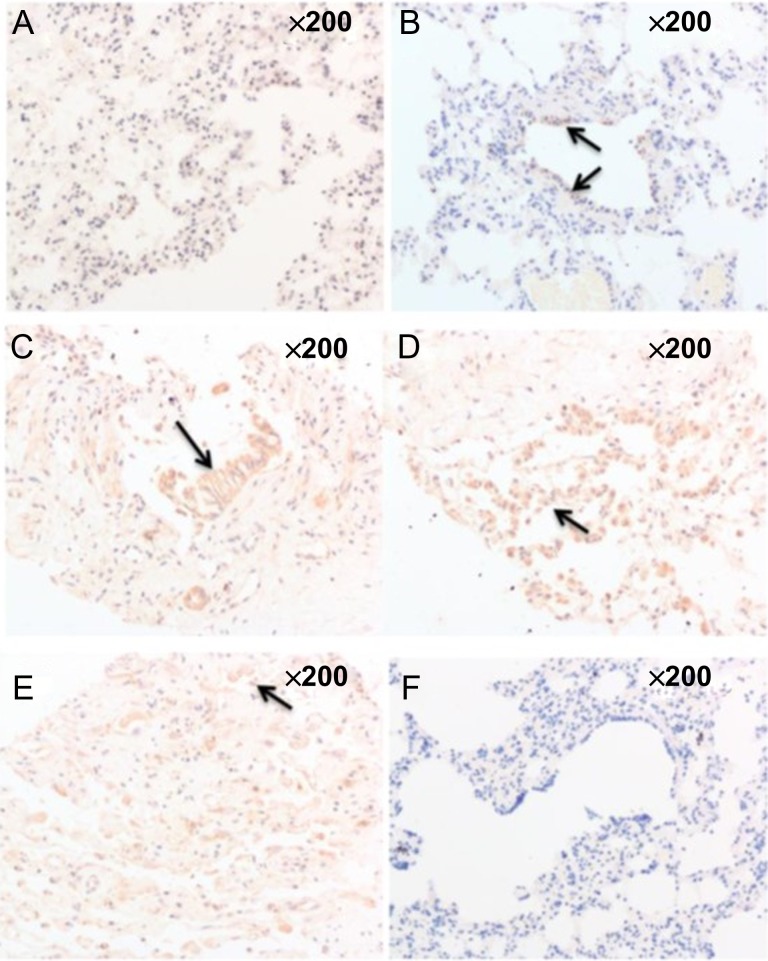
Dynamic changes in MnSOD protein expression in lung tissues of beagles with radiation-induced lung injury: (A) the lung tissues of the non-irradiated group showed almost no expression of MnSOD; (B) 4 weeks after radiation, MnSOD was mainly expressed in the bronchial epithelium; (C) 8 weeks after radiation, MnSOD was still mainly expressed in the bronchial epithelium; (D) 8 weeks after radiation, MnSOD was mainly expressed in the Type II alveolar epithelium; (E) 24 weeks after radiation, MnSOD expression was decreased and was primarily expressed in Type II alveolar epithelial cells; (F) negative control group.

MnSOD is expressed in the bronchial epithelium and Type II alveolar epithelial cells in lung tissues. The cells appear yellow-brown or brownish yellow after immunological staining, and in this case, the nuclei were not stained. The lung tissues of the non-irradiated group showed almost no expression of MnSOD (Fig. 3A), whereas the MnSOD expression levels in the lung tissues of beagles in the irradiated groups increased and changed dynamically with time. MnSOD was mainly expressed in the bronchial epithelium 4 weeks after radiation exposure (Fig. 3B). Its expression peaked at 8 weeks after exposure and was primarily expressed in bronchial epithelium and in Type II alveolar epithelium (Fig. 3C and D). Twenty-four weeks after irradiation, MnSOD expression was decreased compared with that at 8 weeks and was primarily expressed in Type II alveolar epithelial cells (Fig. 3).

### Dynamic changes in the MDA content and reductase activity in the peripheral blood of beagles with radiation-induced lung injury

**Fig. 4.**
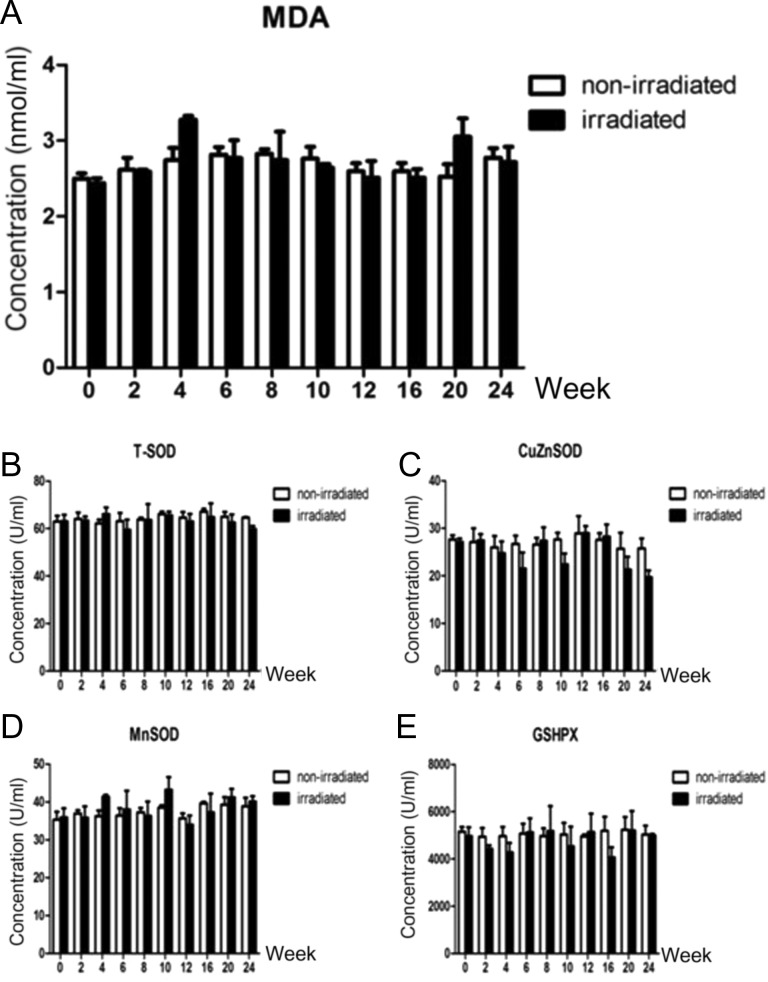
(A) Dynamic changes in the MDA content in the peripheral blood of beagles with radiation-induced lung injury. (B–E) Dynamic changes in the reductase activity in the peripheral blood of beagles with radiation-induced lung injury: (B) dynamic changes in T-SOD enzyme activity; (C) dynamic changes in CuZnSOD enzyme activity; (D) dynamic changes in MnSOD enzyme activity; (E) dynamic changes in GSHPX enzyme activity.

The levels of MDA in the plasma in the irradiated and non-irradiated groups were measured at 0, 2, 4, 6, 8, 10, 12, 16, 20 and 24 weeks after irradiation. The MDA content in the irradiated group was increased at the 4th and 20th weeks (Fig. 4A), but the activity of T-SOD was stable at each time point in the irradiated and non-irradiated groups (Fig. 4B); the activity levels of CuZnSOD at the 6th, 10th, 20th and 24th weeks in the irradiated group were lower than those in the control group (Fig. 4C). The MnSOD activity levels in the irradiated group were higher than those in the control group at the 4th and 10th weeks. At the 16th week, the MnSOD enzyme activity was higher than that of the control group (Fig. 4D). The GSHPX enzyme activity in the irradiated group was lower than that of the control group at Week 16 (Fig. 4E). However, none of the above changes were statistically significant.

### Changes in the copy number of mtDNA in the lung tissues of beagles with radiation-induced lung injury

**Fig. 5.**
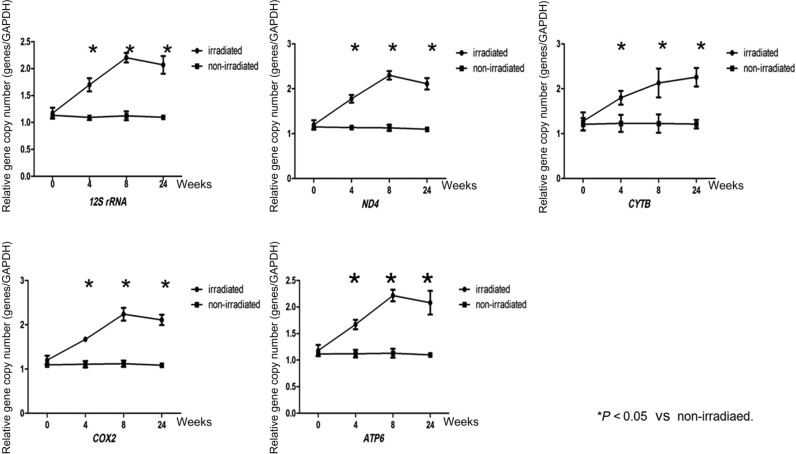
Changes in the copy number of mtDNA in the lung tissues of beagles with radiation-induced lung injury. The copy numbers of the *12S rRNA*, *ND4*, *Cox2* and *ATP6* genes peaked at 8 weeks after irradiation and then decreased at 24 weeks, whereas the copy numbers of the *Cytb* gene increased continuously and peaked at 24 weeks after irradiation. The copy numbers of mitochondrial genes in the lungs of the control group did not differ from one time point to another. Three independent biological replicates were used. *P < 0.05.

The copy numbers of the *12S rRNA*, *ND4*, Cytb, *Cox2* and *ATP6* genes were all increased in irradiated lung tissues at 4, 8 and 24 weeks after irradiation, and the copy numbers of the *12S rRNA*, *ND4*, *Cox2* and *ATP6* genes peaked at 8 weeks after irradiation and then decreased at 24 weeks, whereas the copy numbers of the *Cytb* gene increased continuously and peaked at 24 weeks after irradiation. All the differences were statistically significant (*P* > 0.05). As shown in Fig. 5, the copy numbers of mitochondrial genes in the lungs of the control group did not differ between each time point.

### Changes in the functions of the respiratory chain in lung tissue

**Fig. 6.**
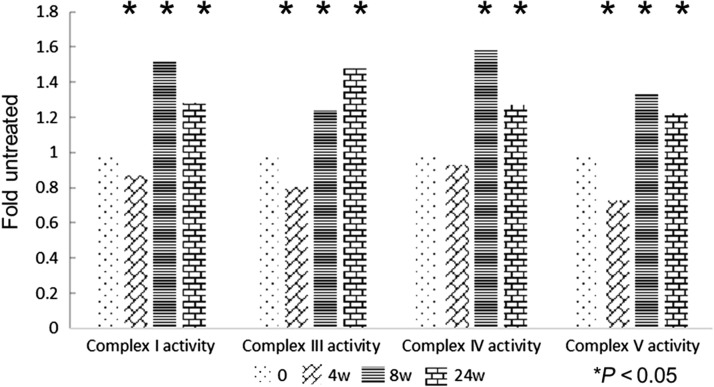
The activity levels of Complex I, III, IV and V exposed to irradiation compared with the activity levels in sham-irradiated mitochondria. The changes in the activity levels of respiratory chain complexes I, IV and V first declined (after 4 weeks), and then peaked at 8 weeks. The activity level of respiratory chain complex III did not peak until 24 weeks after irradiation. Three independent biological replicates were used. *P < 0.05.

The changes in the functions of respiratory chain complexes I, IV and V and the changes in copy number of the corresponding mtDNA-encoded genes were similar in the tissues at 4, 8 and 24 weeks after radiation exposure; the changes were the greatest at 8 weeks and then displayed a decreasing trend until 24 weeks. The difference was that the copy numbers of mtDNA-encoded genes were increased at each time point after irradiation, but the respiratory chain complex function first declined (after 4 weeks) and then increased. The function of the respiratory chain complex III did not peak until 24 weeks after irradiation, which was also the case with the corresponding gene *Cytb* (Fig. 6).

## DISCUSSION

Mice, rats, canines and rhesus macaques have always been selected as experimental animal models for RILI research. Mice and rats are too small to accurately expose the organ to radiation. For example, when the unilateral lung is established as the target organ, smaller animals always receive whole-body radiation, which can elicit a general reaction in multiple organs rather than specifically in the lung tissue. The same experiment can be performed on rhesus macaques; however, this procedure may be too expensive. Therefore, beagle dogs were the best choice [13].

The purpose of this study was to examine the biological changes at different stages of lung injury in beagles after exposure to radiation. A single dose of 18 Gy radiation administered to the right 2/3 of the lungs of beagles can be applied to establish a stable animal model of RILI. No significant changes were observed in body temperature, body weight, mental status, food or water intake, or urination or defecation habits in beagles during the 40-week observation period compared with the dogs in the non-irradiated group. This suggested that the radiation doses and areas were well tolerated by the animals, which allowed continued observation of changes over an extended period of time.

The findings of the CT scan demonstrated that the imaging characteristics obtained from early and late imaging [13] of RILI in the dogs were quite similar to those that are observed during the occurrence and development of RILI in humans [19–21]. Two distinct clinical, pathologic, and radiologic phases of RILI are recognized: an early phase of radiation pneumonitis, which usually occurs between 1 and 6 months after the completion of radiation therapy; and a later phase of chronic radiation fibrosis, which usually occurs between 6 and 12 months after the ompletion of radiation therapy [22]. Among the various end points, radiotherapy-induced radiologic abnormalities can be chosen and quantified as changes in tissue density with CT in our clinical work [23]. These characteristics include ‘frosty-glass’ changes in the tissues, increased shadows that indicate patchy densities, linear shadows and localized emphysema at the irradiated area. Based on the radiation-induced pathological changes observed in animal experiments, Travis divided RILI into three stages: interstitial pulmonary edema, alveolar inflammation and interstitial fibrosis [24]. With these stages in mind, we chose to examine tissues at 4 weeks, 8 weeks and 24 weeks after irradiation as time points for observation. Using CT-guided biopsy to obtain samples, we could examine the pathological changes and corresponding biological changes in lung tissues.

In recent years, radiation-induced tissue changes and cellular oxidative stress have received increased attention. After they were irradiated with 10 Gy 10 MV X-rays, PC-3 human prostate cancer cells showed increased ROS production, with ruptured lysosomes and fragmented mitochondria. Additionally, the rate of apoptosis of these cells was significantly higher than that of the control cells [25]. Similar phenomena were also observed in animal models [26]. ROS interact with DNA and cause DNA damage via the formation of 8-OHdG [27]. Our study examined the dynamic changes of ROS content *in situ* in lung tissues and showed the important role of ROS in the development of RILI. The amounts of tissue superoxide anion, hydrogen peroxide and hydroxyl radicals *in situ* in lung tissues were also increased at 4 weeks, and at that time they peaked. At this time point, only a small number of frosty glass-like changes and patchy density increases were observed by CT imaging, which suggests that the content change of ROS may be a more sensitive marker than that detected by imaging technology. The increase in ROS may be related to the infiltration of inflammatory cells and the secretion of cytokines [28].

The expression levels of MnSOD are closely related to the level of cellular radiation damage. Epperly *et al.* administered MnSOD liposomes or adenoviral vectors into the tracheas of mice. This approach significantly reduced the expression of IL-1, TNFα and TGFβ mRNA and consequentially prevented acute radiation pneumonitis and chronic radiation-induced pulmonary fibrosis [29]. The downregulation of MnSOD expression by RNA interference (RNAi) or induction of mutations reduces the body’s ability to fight oxidative stress and thus aggravates radiation damage [30]. In mouse models of lung injury, it has been confirmed that both the number of mitochondria and the expression of MnSOD are selectively increased in alveolar Type II epithelial cells and vascular endothelial cells [10]. We are interested in the exact intrinsic function of MnSOD during the development of RILIs. It was shown that MnSOD is primarily expressed in the bronchial epithelium 4 weeks after radiation exposure, and that this expression peaks at 8 weeks. This process is slower than the increase in ROS content, which suggests that elevated ROS content in lung tissue may promote MnSOD expression. When oxidative stress increases within the body, a compensatory high expression of MnSOD is required to remove the excess ROS. When the ROS content in tissues drops, the signal to stimulate the expression of MnSOD weakens, and the expression of MnSOD decreases. Current research suggests that the occurrence of RILI is associated with damage to vascular endothelial cells and Type II alveolar epithelial cells. Type II alveolar epithelial cells synthesize and secrete surfactants to maintain alveolar surface tension. Radiation acts directly or indirectly to damage Type II alveolar epithelial cells and causes the release of surfactants, thereby altering the alveolar surface tension and causing further changes in the blood perfusion of lung tissues. At the same time, the cells display damage to the lysosomes, mitochondria, endoplasm reticula and other organelles, which initiates a series of cascades mediated by cytokines. These cascades eventually lead to sclerosed alveolar walls, decreased lung compliance and a reduced number of endothelial cells [31]. In this study, the elevated expression of MnSOD in Type II alveolar epithelial cells and bronchial epithelial cells suggests that MnSOD may play a protective role in RILI. This protective effect manifests in bronchial epithelial cells in the early stages of the injury and in Type II alveolar epithelial cells in the late stages of the injury.

In addition to local oxidative stress, oxidative stress at the circulatory level in organisms should also be noted. Because the peripheral blood is more easily available in clinical practice, the detection of changes in oxidative stress in peripheral blood has a certain value and significance. GSHPX is a reductive enzyme that is widely present in the body. This enzyme catalyzes the reduction of H_2_O_2_ to H_2_O. The measurement of GSHPX activity can be used as a biochemical marker of the antioxidant capacity of tissues [32]. By attacking polyunsaturated fatty acids in biomembranes, ROS triggers lipid peroxidation, which results in the formation of MDA. Therefore, MDA content can reflect the level of lipid peroxidation by ROS in cells [33]. Interestingly, we have not found a significant change in the content of MDA or in the activities of MnSOD, CuZnSOD, T-SOD and GSHPX in peripheral blood, which suggests that the occurrence and development of RILI are closely related to the changes in localized oxidative stress in lung tissues, but not to the state of oxidative stress in the circulation. These findings indicate that RILI may result only from a localized change in oxidative stress in lung tissues. Compared with peripheral blood, the local oxidative stress within lung tissues can better reflect the occurrence and development of RILI.

Some researchers have shown that radiation can induce DNA damage and mutations in mitochondria [34] and can reduce mitochondrial RNA transcription [35]. On the contrary, others have suggested that mtDNA damage and gene mutations are potential markers of radioactive damage [36]. In our study, as target genes for observation, we have selected five mtDNA-encoded genes whose products participate in different respiratory chain functions. The copy numbers of all five mtDNA-encoded genes in the radiation group were increased, and among them, four genes (*12S rRNA*, *ND4*, *Cox2* and *ATP6*) peaked at the 8th week after radiation and then decreased at 24 weeks. The copy number of *Cytb* continued to rise until its peak at 24 weeks after radiation. This indicates that mitochondria play distinct roles in different stages of lung injury. In the early stages of RILI (4 weeks), the copy numbers of mtDNA-encoded genes may increase to compensate for the DNA damage, and this compensation effect peaks at 8 weeks. Afterwards, due to the development of pulmonary fibrosis, the copy numbers of mtDNA-encoded genes declined again. The changes in the different functional regions of mtDNA are also not uniform. The copy number of the *Cytb* gene does not decrease with tissue fibrosis, but instead, it continues to rise. Does this indicate that different functional regions of mtDNA play different roles in the evolution of RILIs? To test this hypothesis, we examined the functions of the corresponding mitochondrial respiratory chain complexes I, III, IV and V, where the *ND4*, *Cytb*, *Cox2* and *ATP6* genes, respectively, work. The results showed that even when the copy numbers of mtDNA-encoded genes increased compensatorily at Week 4, the functions of the respiratory chain showed a decreasing trend, which suggests that the compensatory raise in mtDNA was not normal. At 8 weeks, the functions of the respiratory chain began to increase, but they declined at 24 weeks. Coincidentally, unlike other respiratory chain complexes, the copy numbers of respiratory chain complex III and the associated gene *Cytb* both peaked at 24 weeks. Therefore, different functional regions of mtDNA may play different roles in RILI, but more systematic studies are needed to define the specific mechanism. The mechanisms of duplication and degradation of mtDNA are quite complicated and involve the nuclear genes, mitochondrial transcription factor A [37, 38] and *p53* [39], among others. Furthermore, the roles that these genes play in the process of increasing mtDNA content remain to be determined.

## CONCLUSIONS

RILI is a dynamically changing process. Its occurrence is mainly related to the interaction between local ROS and MnSOD. Mitochondria and their respiratory chain complexes are involved in the overall process of RILI. Various mitochondrial functional regions may play different roles, but the many different mechanisms involved in this process require further investigation.
